# Dietary Fiber Intake and Weight Status in Young Austrian Adults

**DOI:** 10.3390/foods14223863

**Published:** 2025-11-12

**Authors:** Maria do Carmo Greier, Jozsef Dudas, Klaus Greier, Markus Posch, Benedikt Gabriel Hofauer

**Affiliations:** 1 Department of Otorhinolaryngology, Head and Neck Surgery, Medical University of Innsbruck, 6020 Innsbruck, Austria; 2 Department of Sports Science, Leopold-Franzens University Innsbruck, 6020 Innsbruck, Austria; 3 University College of Education (KPH) Stams, 6422 Stams, Austria

**Keywords:** gut microbiota, body mass index, diet quality, microbiome, overweight

## Abstract

Background: Dietary fiber plays a crucial role in body weight regulation and metabolic health. Despite benefits, fiber intake remains suboptimal. This study investigated the relationship between dietary fiber intake, BMI, and fiber sources among young Austrian adults in higher education. Methods: A cross-sectional study was conducted using a validated screening tool (18-item FiberScreen) to assess total daily fiber intake. Self-reported anthropometric data were used to calculate BMI, and participants were categorized into normal-weight and overweight groups. Differences in fiber intake and sources were analyzed by sex and BMI category. Correlations between fiber intake, BMI, and food group contributions were assessed. Results: The mean daily fiber intake among participants (n = 813) was 15.72 g/day. Overweight individuals consumed significantly less total fiber (13.18 ± 0.44 g/day) compared with those of normal weight (16.09 ± 0.22 g/day). Normal-weight participants obtained fiber mainly from fruits, vegetables, and legumes, while the overweight group relied more on potatoes and white bread. Fiber intake was also negatively correlated with BMI (*p* < 0.001). Conclusions: Low fiber intake is widespread among young adults and associated with higher BMI. The findings suggest that not only the quantity but also the quality of fiber sources play a role in weight status. Interventions aimed at improving dietary fiber intake through targeted education and healthier food environments would be a good strategy to support better health outcomes in young adults.

## 1. Introduction

Fiber is a vital component of a healthy diet and plays a foundational role in supporting many aspects of human health [1]. Dietary fiber is a type of carbohydrate and is mostly found in plant-based foods such as fruits, vegetables, legumes, whole grains, nuts, and seeds. There are two main types of fiber: soluble and insoluble. Soluble fiber dissolves in water and helps to regulate blood sugar levels and lower cholesterol, while insoluble fiber adds bulk to stool and supports regular bowel movements, preventing constipation and promoting digestive regularity [2]. However, instead of being broken down and absorbed like other nutrients, dietary fiber cannot be digested by human enzymes. This resistance to digestion is precisely what makes it such a powerful contributor to health.

The gut microbiome is a central regulator of not only digestive function but also immune health, metabolism, inflammation, and even neurological and emotional well-being [3,4]. Fiber acts as a prebiotic, serving as the primary fuel source for many beneficial gut bacteria. When fermentable fibers, such as inulin, resistant starches, and certain non-digestible polysaccharides, reach the colon, microbes, through fermentation, metabolize them. Through this fermentation, different metabolites, like, for example, short-chain fatty acids (SCFAs), are produced [3,5]. These SCFAs play vital roles in reducing systemic inflammation, modulating immune responses, enhancing the gut barrier, and supporting insulin sensitivity [6]. Therefore, fiber is not just a passive dietary component but also a modulator of human health. Scientific research over the past two decades has demonstrated that fiber intake is associated with a significantly lower risk of several chronic diseases, including type 2 diabetes, cardiovascular disease, colorectal cancer, and obesity [3,6]. Furthermore, the composition and activity of the gut microbiota, supported by dietary fiber, have been linked to better mental health, hormonal regulation, and protection against autoimmune and neurodegenerative conditions [7]. In contrast, diets low in fiber, which are common in Western countries, can reduce microbial diversity and promote microbial dysbiosis and increased inflammation [8]. This can cause many health problems, including digestive issues, metabolic diseases, and even psychological disturbances [4].

Despite the extensive evidence of its benefits, most people do not meet the recommended daily fiber intake, which is around 25 to 30 g per day [3,9,10]. Regional guidelines from the European Food Safety Authority (EFSA) and the D-A-CH also recommend similar guidelines for adults, suggesting around 25–35 g of fiber per day [11,12]. Many consume less than half of these amounts, contributing to what is now referred to as the “fiber gap” [13]. This deficiency is linked to rising rates of chronic diseases, systemic inflammation, and weakening of the gut–immune interface [6]. Prioritizing fiber-rich foods can enrich the gut microbiome, support immune resilience, enhance metabolic function, and reduce the risk of disease across the lifespan. Moreover, low fiber intake is also strongly associated with an increased risk of overweight and obesity, conditions that are now widespread across the globe [14].

Obesity and overweight have become a serious global health concern, affecting more than one billion people across all age groups, contributing to rising rates of chronic conditions such as type 2 diabetes, cardiovascular disease, fatty liver disease, certain cancers, and overall reduced life expectancy [15]. While genetic and behavioral factors contribute, the primary driver behind this trend is the increasingly poor quality of modern diets. A reduction in traditional, whole-food-based diets and an increase in diets high in ultra-processed foods, added sugars, unhealthy fats, and refined starches promote excessive calorie intake, metabolic imbalance, and long-term weight gain [16]. These energy-dense but nutrient-poor diets increase the risk of obesity and lead to a paradoxical form of malnutrition. Many people with overweight and obesity suffer from micronutrient deficiencies, despite consuming more calories [17]. This form of malnutrition impairs immune function, lowers energy levels, and influences metabolic health. Aggravating this problem, essential components of a healthy diet, such as fruits, vegetables, legumes, whole grains, and nuts, are often missing or not eaten enough [1]. These foods provide key vitamins, minerals, and dietary fiber that are important not only for metabolic balance but for preventing disease [18]. Overall, the obesity crisis is not just about individual behavior. It is driven and influenced by larger problems in the global food system and industries, including heavy marketing of processed foods and limited access to affordable, nutritious options. Moreover, inadequate public health education and the lack of information about healthy eating worsen this problem [18,19]. The focus needs to change from just reducing calories or losing weight to eating healthier overall. This means making access to nutritious, mostly natural foods easier and changing food systems and their rules that affect daily eating habits [20]. Without action, the health and social problems caused by obesity will keep getting worse in the coming years.

Dietary fiber plays a key role in both the prevention and management of overweight and obesity, with its related health complications [21]. New evidence suggests that fiber-rich diets not only help regulate body weight but also target several biological mechanisms involved in energy balance and metabolism [22]. Fiber can influence satiety and calorie intake by promoting a feeling of fullness, delaying gastric emptying, and reducing overall hunger, which helps prevent overeating and supports weight management efforts [23,24].

Moreover, fiber positively affects gut health, which is closely linked to obesity through the gut microbiome. SCFAs, for example, produced by microbiota through fermentation of fiber, play a crucial role in regulating energy metabolism, reducing low-grade inflammation, and improving insulin sensitivity. These are all key biological factors involved in obesity and metabolic syndrome [21,25]. Additionally, SCFAs may influence hormones that regulate appetite and fat storage, providing another pathway through which fiber contributes to body weight regulation [23]. Beyond these effects, dietary fiber also improves lipid profiles by lowering serum LDL cholesterol and triglycerides, thus addressing dyslipidemia often seen in overweight individuals. Furthermore, fiber intake helps modulate blood glucose levels by slowing carbohydrate absorption, which reduces postprandial glucose spikes and contributes to improved insulin resistance [26]. Evidence also supports the link between increased fiber intake and weight management. For example, participants in diet programs that focus on high fiber intake often lose weight and see better results in their metabolism [27]. Observational studies also suggest that individuals with higher fiber intakes are less likely to be overweight or obese compared with those with low fiber consumption [28]. Moreover, fiber supplements have also demonstrated effectiveness in weight control, especially when combined with lifestyle changes, highlighting their potential as part of therapeutic strategies for obesity [25]. Fiber is a powerful tool against overweight and obesity and an essential part of prevention and therapy [28,29]. As obesity rates and obesity-related diseases continue to rise worldwide, increasing fiber intake should be considered a key component of dietary strategies for weight management and health improvement [21,22].

Therefore, this study aimed to assess the connection between daily dietary fiber intake and weight status in adolescents, contributing to a deeper understanding of factors that may increase overweight and obesity.

## 2. Materials and Methods

The present study used a cross-sectional design to assess dietary fiber intake among young adults in higher education. Therefore, the 18-item FiberScreen questionnaire was used. The survey was directed at students from six different higher education institutions located across four federal states in Austria (Tyrol, Vorarlberg, Lower Austria, and Upper Austria). Data collection was conducted over a five-week period (5 May 2025 until 8 June 2025). The questionnaire was created and distributed online via Microsoft Forms through email and a QR code. To maximize the response rate, a reminder email was sent to all participating institutions at the end of the third week.

Data on participants’ average daily fiber intake as well as demographic information, including age, gender, and body mass index (BMI), were collected. Both dietary intake and anthropometric data (height and weight) were self-reported by participants. As these measures were self-reported, there was a possibility of small systematic errors, such as a slight underestimation of weight or an overestimation of height, which could, in turn, affect BMI calculations. Gender was recorded in binary format (male/female), and age was provided in years. All students enrolled at the participating institutions who voluntarily completed the questionnaire during the data collection period were included. Thus, this study used a voluntary, self-selected sample, including all students who chose to participate during the data collection period. The inclusion criteria were having a minimum age of 18 years and being currently enrolled in the participating institutions. No participants were excluded based on clinical conditions, medication use, or special groups (e.g., professional athletes), as all students who voluntarily completed the questionnaire were included. Participants were excluded if they did not provide complete dietary or anthropometric data.

This study received ethical approval from the Board for Ethical Questions in Science of the University of Innsbruck prior to the data collection (Certificate of Good Standing, 18/2025). Participation was voluntary and anonymous, and participants were informed about the purpose, procedures, data protection measures, and that by completing the questionnaire, they consented to the use of their responses for research purposes.

### 2.1. FiberScreen Questionnaire

The 18-item FiberScreen questionnaire is a validated dietary assessment tool developed by experts at Wageningen University and Research, designed to efficiently estimate habitual dietary fiber intake in adults [30]. It covers a broad spectrum of fiber-rich food categories, including whole grains, fruits, vegetables, legumes, nuts, and seeds. Each item in the questionnaire targets one food group and consists of closed-ended questions: one asking about the frequency of consumption (e.g., how many days per week the food is typically consumed) and another asking about the typical amount consumed per day (e.g., number of portions, slices, or serving spoons). Participants report their average intake based on the previous week. The responses are structured using categorical scales to reflect common consumption patterns in a standardized manner. To estimate fiber intake, participant responses are converted into daily fiber intake (in grams) using a scoring algorithm that multiplies the reported frequency and quantity by specific fiber content factors assigned to each food category. These fiber factors are based on the typical fiber content per portion and were provided as part of the validated scoring syntax developed by Wageningen University. In this study, a German-translated version of the original FiberScreen was used. The translation was reviewed and corrected by a bilingual professor (German and English) and pilot-tested with 30 students to ensure clarity. The pilot test was conducted solely to ensure the translation was understandable and culturally appropriate, without formal assessment of reliability or validity. The translation retained the structure and scoring framework of the original tool and was adapted for clarity and cultural relevance. As this questionnaire is proprietary and must be purchased from the developers, it cannot be freely shared or attached to this manuscript.

### 2.2. Data Analysis

Data analysis of the 18-item FiberScreen questionnaire was conducted using the syntax provided by the questionnaire developers to calculate total and category-specific fiber intake in grams per day. This syntax incorporates standardized conversion factors, frequency weights, and portion estimates, ensuring consistent and reproducible scoring across participants. Descriptive statistics were used to present the means and standard errors of the measurements. All graphs are presented as means ± SEMs. Our data were normally distributed, which was assessed using the D’Agostino–Pearson omnibus normality test. Differences between means were compared with Student’s *t*-test, at a confidence interval of 95%. The relationships of scaled measured values were tested by Pearson correlations, the correlation coefficients and the *p*-values were shown at a 95% confidence interval. A power analysis with a target statistical power of 0.8 was performed to determine the required sample size. For this, the actual mean and standard deviation values of groups 1 and 2 were added into the tool provided by SPSS Version 30. The power/sample size analysis was bidirectional, since differences in both directions were relevant. Our sample size was clearly higher. Considering all the data presented, the statistical power was more than 0.8 at the available number of participants. For the effects of ordinal parameters on the BMI-scaled values, a generalized linear model was used, assuming a gamma distribution and a logistic link function. Main effects and interactions were included. Parameters were estimated using the maximum likelihood method, and *p*-values and confidence intervals were calculated according to Wald. All calculations were performed with SPPS Statistics Ver. 30 (IBM, Armonk, NY, USA). Estimated marginal means (EMMs) and their standard errors (SEMs) were graphically presented using GraphPad Prism 10 (GraphPad Software, San Diego, CA, USA).

## 3. Results

All available participants were included in the final analyses. A total of 813 participants were included in this study, of which 96 were male and 717 were female. The average age was 22.34 (Table 1). Power calculations indicated that a considerably smaller number of participants would have been sufficient to achieve a statistical power of 0.8 at a 95% confidence level. Because the actual sample size was substantially larger than this calculated minimum, the statistical power for all reported analyses exceeded 0.8.

### 3.1. Fiber Intake

**Figure 1:**
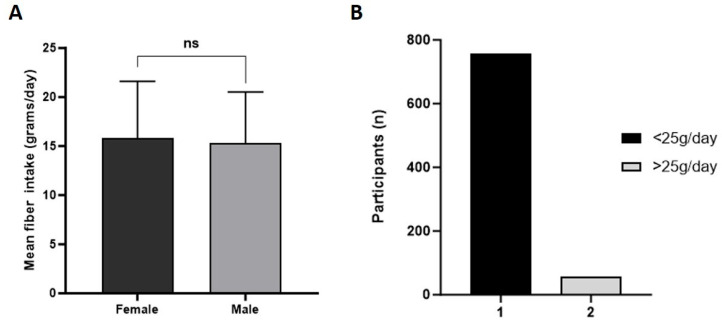
Daily total fiber intake. (**A**) Mean daily fiber intakes of female and male students (error bars: SEMs; ns = not significant) (**B**) Daily fiber intake in categories one and two (1: <25 g/day; 2: >25 g/day).

With the FiberScreen questionnaire, the total daily fiber intake of 813 students was assessed. Overall, the mean daily fiber intake was 15.72 ± 0.203 g/day, with a minimum daily fiber intake of 3.99 g per day and a maximum intake of 47.47 g per day (Figure 1). The mean daily fiber intake did not differ between male and female participants (*p* = 0.349; Figure 1A). Subgroup analyses of fiber quality, however, revealed sex-specific patterns. Higher vegetable fiber intake among normal-weight females accounted for much of the overall difference between BMI groups, while fiber from dried fruit and nuts was significantly associated with BMI only in females. In males, vegetable (*p* = 0.10) and potato (*p* = 0.16) fiber intake did not differ between normal-weight and overweight participants, whereas white-pasta fiber intake showed a modest but significant difference (*p* = 0.023; 95% CI: −0.465 to −0.0037).

The WHO recommends ingesting a minimum of 25 g of fiber per day. Therefore, fiber intake was categorized into two groups. Group 1 represented a daily fiber intake under 25 g per day; group 2 represented a consumption of over 25 g of fiber per day. The data indicate that 93.1% (n = 757) of the students consumed under 25 g of fiber per day. Only the minority of 6.9% (n = 56) ingested over 25 g of fiber per day (Figure 1B). The majority of females (n = 699) and males (n = 95) did not meet current recommendations. Moreover, correlations between age and fiber intake were also analyzed; thus, there was no significant correlation between age and daily fiber intake (*p* = 0.758).

**Figure 2:**
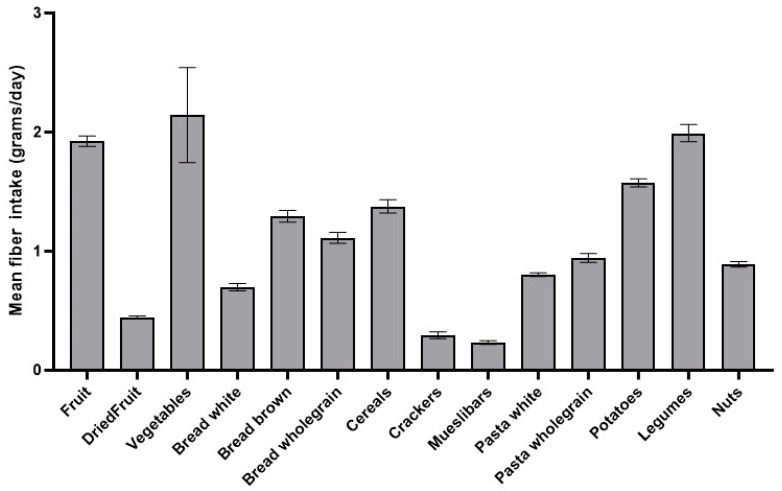
Mean daily fiber intake (g/day) per food category. Highest amount of fiber was achieved by consumption of vegetables, legumes, fruit, and potatoes, and lowest by consumption of muesli bars and crackers (error bars: SEMs).

To find out which food groups contributed most to daily fiber intake, the mean fiber intake (g/day) was analyzed per food category (Table 2 and Figure 2). The results indicated that most of the participants obtained their fiber content through vegetables (2.15 ± 0.04 g), followed by legumes (1.99 ± 0.07 g), fruit (1.92 ± 0.05 g), and potatoes (1.57 ± 0.03 g). The lowest fiber intake was achieved through muesli bars (0.23 ± 0.02 g) and crackers (0.30 ± 0.03 g) (Table 2; Figure 2).

### 3.2. Weight Status and Fiber Intake

**Figure 3:**
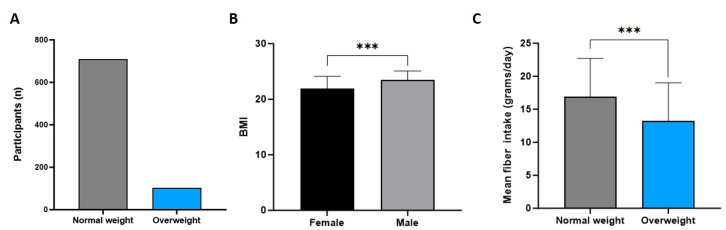
Weight status of participants and mean fiber intake. (**A**) Number of normal-weight and overweight participants. (**B**) Mean BMIs of females and males. (**C**) Mean fiber intakes (g/day) of normal-weight and overweight participants. (Error bars: SEMs; *** *p*-value: <0.001).

To analyze weight status, their BMI was calculated from the height and weight stated in the questionnaire. Of 813 students, 710 (87.3%) were of normal weight with a BMI between 18.5 and 24.9 kg/m^2^. The rest of the students (n = 103; 12.7%) were classified as overweight with a BMI over 25 kg/m^2^ (Figure 3A). The mean BMI for all participants was 22.14 ± 0.15 kg/m^2^; for females, it was 21.97 ± 0.08 kg/m^2^; and for males, it was 23.47 ± 0.17 kg/m^2^ (Figure 3B). The results indicated a significant difference in BMI between females and males (*p* < 0.001), indicating a higher BMI for males compared with females.

**Figure 4:**
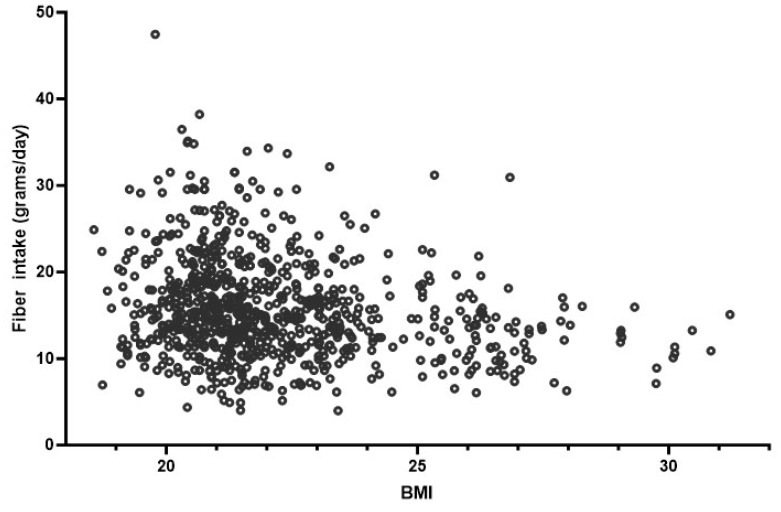
Correlation between daily fiber intake and BMI. Participants with a higher BMI had a lower fiber intake compared with participants with a lower BMI.

Fiber intake also differed between normal-weight (BMI: 18.5–24.9 kg/m^2^) and overweight (BMI ≥ 25 kg/m^2^) participants. The mean fiber intake of normal-weight participants was 16.09 ± 0.22 g/day, and for overweight participants, it was 13.18 ± 0.44 g/day (*p* < 0.001; Figure 3C). Separate analyses by sex showed that normal-weight males consumed significantly more fiber than overweight males (mean difference: −3.33 ± 0.24 g; 95% CI: −3.80 to −2.86; *p* < 0.001), and normal-weight females consumed significantly more fiber than overweight females (mean difference: −5.54 ± 0.14 g; 95% CI: −5.82 to −5.26; *p* < 0.001). These results indicate that the inverse relationship between BMI and fiber intake was consistent within each sex group. Moreover, the results also indicated a significant negative correlation between BMI and daily fiber intake (negative correlation: r = −0.217; *p* < 0.001), indicating that participants with a higher BMI ingested less fiber than participants with a lower BMI (Figure 4).

**Figure 5:**
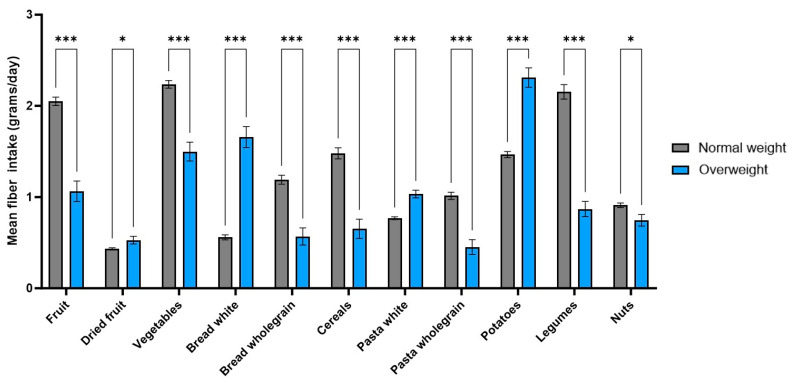
Mean fiber intake through different food categories of normal-weight and overweight participants (error bars: SEMs; * *p*-value: <0.05; *** *p*-value: <0.001).

Since BMI and total fiber intake were found to be associated, fiber intake was further analyzed by specific food categories to assess whether the sources of dietary fiber differed between participants with a normal weight and those who were overweight. The mean fiber intake from each of 14 food categories was compared between the two BMI groups. Significant differences were observed in 11 out of the 14 categories, indicating that the dietary sources contributing to fiber intake varied between normal-weight and overweight individuals (Figure 5; Table 3).

**Figure 6:**
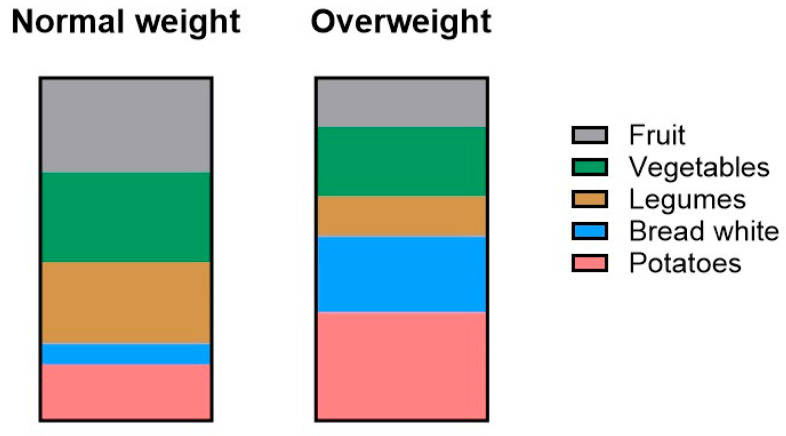
Distribution of mean fiber intake in five food categories between normal-weight and overweight participants.

Significant differences in fiber intake between normal-weight and overweight participants were observed in the following 11 food categories: fruit, dried fruit, vegetables, white and wholegrain bread, cereals, white and wholegrain pasta, potatoes, legumes, and nuts. Among normal-weight participants, the highest contributions to daily fiber intake came from fruits (2.05 ± 0.046 g), vegetables (2.24 ± 0.042 g), and legumes (2.15 ± 0.079 g) (Table 3). In comparison, fiber intake from these same categories was significantly lower in overweight participants (*p* < 0.001). For overweight individuals, the main sources of fiber were potatoes (2.31 ± 0.107 g), white bread (1.66 ± 0.114 g), and vegetables (1.50 ± 0.103 g). The five food categories contributing most to total daily fiber intake in each group were selected and are visualized in Figure 6. While fiber intake from fruits, vegetables, and legumes was higher among normal-weight participants, fiber intake from white bread and potatoes was higher among those who were overweight.

**Figure 7:**
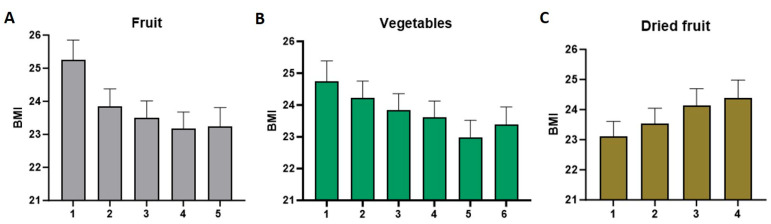
Associations of consumption frequency of fruit, vegetables, and dried fruit with BMI. (**A**) Frequency of fruit consumption (portions/day) in relation to BMI: 1 = I hardly eat any fruit; 2 = less than one portion per day; 3 = 1 portion per day; 4 = 2 portions per day; and 5 = 3 portions or more per day. (**B**) Frequency of vegetable consumption (serving spoons per day) in relation to BMI: 1 = 0 spoons; 2 = 1 spoon; 3 = 2 spoons; 4 = 3 spoons; 5 = 4 spoons; and 6 = more than 4 spoons. (**C**) Frequency of dried fruit consumption (days per week) in relation to BMI: 1 = less than once per week; 2 = 1 to 2 days per week; 3 = 3 to 4 days per week; and 4 = 5 to 7 days per week. (Error bars: SEMs).

To further investigate the relationship between dietary habits and BMI, the frequency of consumption across different food categories was analyzed using a generalized linear model. The frequency of consuming fruit, vegetables, and dried fruit showed a strong and statistically significant association with BMI (all *p* < 0.001; Figure 7).

Participants who reported that they hardly ate any fruit had a higher BMI compared with those who stated eating three or more portions of fruit per day (Figure 7A). Similarly, participants who stated eating no vegetables per day had a higher BMI compared with those who ate more than four serving spoons of vegetables per day (Figure 7B). Interestingly, the pattern was reversed for dried fruit. Participants who ate dried fruit less than once per week had a lower BMI compared with those who ate dried fruit 5 to 7 days per week (Figure 7C).

## 4. Discussion

This study investigated dietary fiber intake among university students, its association with weight status, and the contribution of specific food groups to fiber intake. A total of 813 students were analyzed, revealing that fiber intake was substantially below the current recommendation of 25 to 30 g/day for the vast majority of participants. The results also demonstrated a significant negative correlation between fiber intake and BMI, with overweight participants consuming significantly less fiber than normal-weight participants. Furthermore, differences in the primary sources of dietary fiber between BMI groups were observed, indicating potential dietary patterns that may contribute to weight status among young adults.

### 4.1. Fiber Intake Among Young Adults

Mean daily fiber intake among participants was around 15.7 g/day, which is approximately 48% lower than the recommended daily intake. While the WHO guidelines suggest ≥25 g/day, regional EFSA and D-A-CH recommendations advise 25–35 g/day for adults, highlighting that the participants’ intake was far below both international and regional benchmarks. The majority of the participants consumed less than 25 g/day, and only a small amount met the recommendation of 25 g/day. These findings are concerning but consistent with global and regional trends in young adults’ dietary habits, where fiber intake tends to be low due to high consumption of ultra-processed foods and refined carbohydrates [31,32]. Recent studies in Swiss adults and medical students also report low fiber intake and high consumption of ultra-processed foods, confirming these patterns across European young populations [33,34]. High intake of ultra-processed foods and sugar-sweetened beverages can displace fiber-rich foods such as fruits, vegetables, legumes, and whole grains, reducing overall fiber intake and contributing to higher BMI. Therefore, not only the quantity but also the quality of the overall diet is relevant when assessing fiber intake patterns in young adults. It should also be noted that this study focused on total dietary fiber intake and did not differentiate between soluble and insoluble fibers, which may have distinct effects. The lack of difference in fiber intake between male and female participants suggests that low fiber consumption is a universal issue across genders in this population. Previous studies have also found similar trends in adults, highlighting a general neglect of whole foods rich in dietary fiber, such as fruits, vegetables, and legumes, in favor of more palatable and convenient processed foods [32,35,36]. Similar low intakes were also reported in Slovenia, where most adolescents and adults failed to meet fiber targets; thus, their slightly higher averages likely reflect more legumes and use of full 24 h recalls [37]. A European review also showed most countries reach only 60–70% of the 25–35 g/day goal, with differences explained by national diets and survey methods [38]. Also, in the US, young adults showed comparable shortfalls despite higher total calories, underlining that the problem is global, not just regional [39].

### 4.2. Weight Status and Fiber Intake

Notably, males had a significantly higher BMI than females. More importantly, overweight participants had a significantly lower fiber intake compared with their normal-weight peers. A significant inverse correlation between BMI and fiber intake underscores a potential association between dietary fiber intake and weight status. Although the absolute difference in fiber intake between groups was modest (~3 g/day), this may still be meaningful as a marker of overall diet quality rather than a direct causal effect on BMI. Higher fiber intake likely reflects healthier dietary patterns, such as increased consumption of fruits, vegetables, and whole grains, which collectively contribute to better weight management. Similar inverse associations between fiber intake and BMI have been reported in Slovenian adults, European adolescents, and university students [33,37,40]. These results are supported by numerous studies showing a strong inverse relationship between fiber intake and both BMI and fat accumulation. One study showed that increasing fiber intake significantly reduces the risk of weight and fat gain in women [41]. Another one found that higher fiber consumption predicted greater weight loss and better adherence to calorie-restricted diets, suggesting a potential role for fiber in weight management [42]. Our findings are also consistent with others who reported that increased dietary fiber intake was associated with reductions in both BMI and waist circumference in young adults [35]. Fiber reduces energy density, slows digestion, and enhances satiety, which are all possible mechanisms that may contribute to weight control [32]. Our inverse BMI–fiber link matches findings in overweight adolescents, where higher fiber reduced metabolically unhealthy status, and in another study, where both soluble and insoluble fiber improved insulin sensitivity [42,43]. Older US cohorts also showed that normal-weight adults eat more fiber and fruit than heavier peers, closely reflecting our Austrian data [39,44]. Moreover, our analyses by sex showed that normal-weight participants consumed significantly more fiber than overweight participants in both males and females. It should be noted that the predominance of female participants (88%) may limit generalizability to the overall population of young adults, particularly regarding male-specific BMI and fiber intake patterns. Nevertheless, within this sample, the inverse relationship between BMI and fiber intake was observed in both males and females.

### 4.3. Sources of Fiber and Differences by Weight Status

Our findings revealed differences in the sources of dietary fiber between participants with normal weight and those classified as overweight. While both groups consumed fiber from a variety of food categories, normal-weight participants reported significantly higher intake from fruits, vegetables, and legumes. In contrast, the overweight group obtained most of their fiber from potatoes, white bread, and vegetables, though their intake from vegetables was still lower than that of the normal weight group. This suggests that both the quantity and the quality of fiber sources may play a role in weight status. However, it is worth noting that the fiber reported from potatoes may not reflect purely healthy sources. In the questionnaire, potato intake included various preparations (boiled, baked, mashed, or fried), potentially influencing fiber values through less healthy options like fries and chips. Cooking and processing methods can change the fiber content or its physiological availability, for example, through water absorption, heat, or removal of fibrous parts during preparation. However, the FiberScreen questionnaire does not differentiate between preparation methods for any food category, which could introduce small variations in estimated fiber intake. Since the FiberScreen questionnaire sums all preparation types within one item, more detailed distinctions were not possible and should be kept in mind when interpreting fiber quality.

Other studies showed similar patterns to ours. Davis et al. (2006) found that normal-weight adults ate more fruit fiber, while overweight adults ate more refined grains, which matches our fruit/legume versus potato/white bread results [44]. Another study also noted that bread-based diets like Austria’s may explain why our participants relied more on potatoes and white bread compared with Mediterranean diets [38]. Our findings support the growing evidence highlighting the importance of unprocessed, plant-based sources of fiber in relation to maintaining a healthy weight. Diets rich in fruits, vegetables, legumes, and whole grains are associated with increased satiety, better glycemic control, and improved metabolic health, all of which are factors potentially influencing body weight regulation [45]. This is consistent with other results that found that plant-based dietary patterns contribute to lower body weight and improved weight status across diverse populations [46]. Our data also suggest that overweight individuals may not only consume less fiber overall, but also derive it from lower-quality sources, such as refined starches and processed foods, rather than fiber-rich fruits and legumes. Analyses by sex showed that normal-weight females consumed more vegetable fiber than males or overweight females, and fiber from dried fruit and nuts was associated with BMI only in females. In males, fiber from vegetables and potatoes did not differ between BMI groups, while white-pasta fiber showed a modest but significant difference, indicating that the differences in fiber quality were largely driven by females. Although males had a higher BMI on average, the overall pattern of fiber sources was similar between males and females, suggesting that sex influenced some fiber intake patterns, but the overall effect on fiber quality was limited in this sample. Therefore, nutrition education should emphasize the health advantages of minimally processed, fiber-rich plant foods as part of strategies that may help against excess weight and associated chronic disease risk.

### 4.4. Frequency of Consumption and Associations with BMI

Important associations between the frequency of consuming specific fiber-rich foods and BMI were identified in this study. Participants who reported eating three or more servings of fruit daily had significantly lower BMI values compared with those who ate hardly any fruit. A similar trend was observed for vegetable intake. Those consuming more than four servings of vegetables daily had significantly lower BMI values. These findings suggest the importance of regular consumption of fiber-dense foods in relation to maintaining a healthy body weight.

Interestingly, dried fruit consumption showed a different pattern. Participants who consumed dried fruit five to seven days per week had a higher BMI compared with those who ate it less than once per week. This might be explained through the energy-dense nature of dried fruits and the possibility of overconsumption. Although dried fruits are rich in fiber, they also lack water volume, contain concentrated sugars and calories, which could contribute to weight gain when consumed in large quantities. Additionally, as only a small number of participants reported consuming dried fruit daily, this pattern should be interpreted cautiously, and potential inaccuracies in self-reported intake cannot be excluded. However, this is not the case for fresh fruit, as frequent consumption of whole fruits, rich in fiber and other beneficial nutrients, was associated with lower BMI in adults, supporting the overall conclusion of a clear inverse relationship between fiber intake and BMI [44]. Our results align with the literature reporting that not all fiber-containing foods are the same in their impact on weight and metabolic health [31,47].

### 4.5. Implications and Future Directions

The current findings show that many young adults do not obtain enough fiber in their diets, and that a lower fiber intake may be associated with a higher BMI. Not only the amount of fiber matters, but also what kinds of foods the fiber comes from, and how often they are eaten, seem to play an important role in this association. These results highlight the need for better nutrition education and diet programs that focus not only on eating more fiber, but also on choosing healthier fiber sources like fresh fruits, vegetables, and legumes instead of more processed carbs. Consideration of broader dietary patterns, including consumption of ultra-processed foods and sugar-sweetened beverages, is also important for understanding their combined effect on BMI. It should also be noted that other dietary components, such as fat, sugar, and protein intake, were not assessed in this study and may also influence BMI, so fiber is only one factor among several contributing to weight status. Future research could also include assessment of total caloric intake to better understand how energy consumption interacts with fiber intake and BMI in young adults. The results also show that simple tools like the FiberScreen questionnaire can help spot people who may be at risk for poor diet quality and weight problems. Future research could look at whether eating more of certain high-fiber foods actually helps lower BMI or improve health in young adults. The most effective strategies might be those that combine education with support for behavior change and a better food environment. Recent studies also demonstrate links between fiber intake, metabolome biomarkers, and cardiometabolic health in European adults, highlighting the broader health relevance of improving fiber consumption [48,49].

Our findings add new evidence on fiber intake patterns in young Central European adults, a population rarely studied in this context. European reviews report that mean fiber intakes are generally below 20 g/day, but most surveys do not examine associations with BMI [38]. Other studies in Slovenia and in Poland have assessed fiber intake in relation to BMI, yet data for Austria are lacking [37,50]. By providing this information, our study complements existing Central European research and fills an important gap, offering insights for targeted public health and nutrition interventions among young adults.

### 4.6. Limitations

Although this study offers useful insights into how fiber intake is linked to body weight in young adults, there are some important limitations to keep in mind. First, the data on diet, height, and weight were self-reported, which means they might not be completely accurate. People can forget what they ate or might report healthier habits than they actually have. Additionally, no validation against objective measures such as biomarkers or direct anthropometric assessments was performed, which may affect the accuracy of both dietary and BMI data. Also, because this was a cross-sectional study, we only looked at one point in time. The 18-item FiberScreen questionnaire is a quick and validated tool to estimate fiber intake, but it does not provide a full picture of someone’s diet. It does not include total calories or other nutrients that could affect body weight, such as fat, sugar, or protein intake. Other factors that influence weight, like physical activity, sleep, or stress, were also not measured, which might have impacted the results. Therefore, the observed associations between fiber intake and BMI should be interpreted as correlations rather than causal relationships, since unmeasured factors, such as total caloric intake or lifestyle behaviors, may also contribute. Moreover, participants were not screened for chronic diseases, medication use, or special populations, which may have influenced the observed associations between fiber intake and BMI. These factors may also influence weight status and dietary habits, and their absence should be considered when interpreting the findings. In addition, most of the participants were female, which reflects the well-known gender distribution in primary school teacher education programs, where women are typically overrepresented. This is a common observation for such courses. However, the heavy female predominance (88.2%) limits the extent to which the findings can be generalized to the wider young adult population in Austria. As males represented a smaller subgroup (n = 96) with a higher average BMI, caution is warranted in extrapolating the findings specifically to male students, although our analyses still provide meaningful insights for both sexes. This study was also limited to a few universities in Austria, meaning that the sample is not fully representative of all Austrian young adults. Finally, we did not use biological markers to confirm fiber intake, so our data relied fully on what participants reported.

Despite these limitations, this study has several strengths that support the validity of the findings. The use of the validated FiberScreen questionnaire provides reliable estimates of fiber intake, and the analysis of fiber quality and sources adds novel insights beyond total intake. Furthermore, the patterns we found, such as differences between normal-weight and overweight participants, are also consistent with previous studies, which increases confidence in the results.

## 5. Conclusions

Our study highlights a significant gap in fiber intake among young adults and reveals a clear inverse relationship between fiber consumption and BMI. Normal-weight participants not only consumed more fiber but also sourced it from healthier foods like fruits, vegetables, and legumes, while overweight individuals relied more on lower-quality sources. These findings reinforce existing evidence that both the quantity and quality of dietary fiber are associated with weight management and metabolic health. Fiber should be seen as a core component of a healthy diet. Therefore, promoting greater intake of high-quality, fiber-rich foods through education and supportive environments may offer a simple, effective strategy to improve health outcomes and reduce obesity risk among young adults.

## Figures and Tables

**Table 1:** Total number of participants, sex distribution, and average age.

	n (%)	ø Age
Female	717 (88.2)	22.28
Male	96 (11.8)	22.77
Total	813 (100)	22.34

**Table 2:** Mean daily fiber intake (g/day) per food category (SEM: std. error of mean).

Food Category	n	Mean Fiber Intake (g/Day)	SEM
Fruit	813	1.92	0.045
Dried fruit	813	0.45	0.012
Vegetables	813	2.15	0.040
White bread	813	0.69	0.031
Brown bread	813	1.29	0.050
Bread wholegrain	813	1.11	0.046
Cereals	813	1.37	0.056
Crackers	813	0.30	0.028
Muesli bars	813	0.23	0.015
White pasta	813	0.80	0.015
Wholegrain pasta	813	0.94	0.037
Potatoes	813	1.57	0.034
Legumes	813	1.99	0.072
Nuts	813	0.89	0.023

**Table 3:** Comparison of mean fiber (g/day) intake per food category of normal-weight and overweight participants (SEM: std. error of mean).

	Normal Weight		Overweight		
Food Categories	Mean Fiber	SEM	Mean Fiber	SEM	*p*-Value
Fruit	2.05	0.046	1.06	0.112	<0.001
Dried fruit	0.43	0.012	0.53	0.041	0.029
Vegetables	2.24	0.042	1.50	0.102	<0.001
White bread	0.56	0.027	1.66	0.114	<0.001
Wholegrain bread	1.19	0.049	0.57	0.094	<0.001
Cereals	1.48	0.061	0.65	0.104	<0.001
White pasta	0.77	0.015	1.03	0.041	<0.001
Wholegrain pasta	1.01	0.039	0.45	0.080	<0.001
Potatoes	1.47	0.034	2.31	0.106	<0.001
Legumes	2.15	0.079	0.86	0.083	<0.001
Nuts	0.91	0.024	0.77	0.063	0.017

## Data Availability

The original data presented in this study are available in Zenodo at doi:10.5281/zenodo.16832402.

## Abbreviations

The following abbreviations were used in this manuscript:

BMI	Body Mass Index
SCFAs	Short-Chain Fatty Acids
EFSA	European Food Safety Authority
EMM	Estimated Marginal Mean
SEM	Standard Error of the Mean
